# A meta-validated immune infiltration-related gene model predicts prognosis and immunotherapy sensitivity in HNSCC

**DOI:** 10.1186/s12885-023-10532-y

**Published:** 2023-01-13

**Authors:** Yinghe Ding, Ling Chu, Qingtai Cao, Hanyu Lei, Xinyu Li, Quan Zhuang

**Affiliations:** 1 Transplantation Center, The 3rd Xiangya Hospital, Central South University, Changsha, 410013 Hunan China; 2 Department of Pathology, The 3rd Xiangya Hospital, Central South University, Changsha, 410013 Hunan China; 3 Research Center of National Health Ministry on Transplantation Medicine, Changsha, 410013 Hunan China

**Keywords:** Prognostic model, Head and neck squamous cell carcinoma, Tumor microenvironment, Immune cell infiltration, Immunotherapy sensitivity

## Abstract

**Background:**

Tumor microenvironment (TME) is of great importance to regulate the initiation and advance of cancer. The immune infiltration patterns of TME have been considered to impact the prognosis and immunotherapy sensitivity in Head and Neck squamous cell carcinoma (HNSCC). Whereas, specific molecular targets and cell components involved in the HNSCC tumor microenvironment remain a twilight zone.

**Methods:**

Immune scores of TCGA-HNSCC patients were calculated via ESTIMATE algorithm, followed by weighted gene co-expression network analysis (WGCNA) to filter immune infiltration-related gene modules. Univariate, the least absolute shrinkage and selection operator (LASSO), and multivariate cox regression were applied to construct the prognostic model. The predictive capacity was validated by meta-analysis including external dataset GSE65858, GSE41613 and GSE686. Model candidate genes were verified at mRNA and protein levels using public database and independent specimens of immunohistochemistry. Immunotherapy-treated cohort GSE159067, TIDE and CIBERSORT were used to evaluate the features of immunotherapy responsiveness and immune infiltration in HNSCC.

**Results:**

Immune microenvironment was significantly associated with the prognosis of HNSCC patients. Total 277 immune infiltration-related genes were filtered by WGCNA and involved in various immune processes. Cox regression identified nine prognostic immune infiltration-related genes (MORF4L2, CTSL1, TBC1D2, C5orf15, LIPA, WIPF1, CXCL13, TMEM173, ISG20) to build a risk score. Most candidate genes were highly expressed in HNSCC tissues at mRNA and protein levels. Survival meta-analysis illustrated high prognostic accuracy of the model in the discovery cohort and validation cohort. Higher proportion of progression-free outcomes, lower TIDE scores and higher expression levels of immune checkpoint genes indicated enhanced immunotherapy responsiveness in low-risk patients. Decreased memory B cells, CD8+ T cells, follicular helper T cells, regulatory T cells, and increased activated dendritic cells and activated mast cells were identified as crucial immune cells in the TME of high-risk patients.

**Conclusions:**

The immune infiltration-related gene model was well-qualified and provided novel biomarkers for the prognosis of HNSCC.

## Background

Head and neck squamous cell carcinoma (HNSCC), a common and aggressive malignancy with high morbidity and mortality, is one of the seven most common malignancies. Annually, there are about 800,000 new cases and more than 400,000 deaths worldwide [1]. Early-stage disease (stages I and II) is treated with single-modality surgery or radiotherapy contributing to high cure rates. However, due to the complex anatomy of head and neck, it is difficult to perform surgery. When patients are diagnosed with head and neck cancer, more than 50% of them are in clinical stage III or IV and lose their best chance of operation [2]. This is one of the reasons why the total global survival rate of HNSCC is only 50%. Besides, local recurrence or metastasis also leads to the poor prognosis of HNSCC.

Traditional treatments are not so effective for HNSCC. Even with aggressive therapy, loco-regional and distant recurrences after treatment are common and thus result in poor prognosis [3]. Despite the continuous innovation of treatment methods, there are still problems such as insufficient efficacy and excessive toxicity. With the advent of molecular targeted therapy, it is expected to replace cisplatin chemotherapy due to its less toxicity. The addition of the EGFR inhibitor cetuximab to radiotherapy has been shown to improve the prognosis of HNSCC patients compared with radiotherapy alone [4]. However, several recent studies indicated poor outcomes when cetuximab was given in HPV-associated HNSCC [5, 6]. Since traditional treatments and molecular targeted therapy cannot satisfy the treatment of HNSCC, immunotherapy has gradually attracted public attention. Taking the tumor heterogeneity and immune states of different individuals into account, it is necessary to identify the immune phenotypes of HNSCC to ensure that patients gain the maximum benefit from immunotherapy.

The tumor microenvironment (TME) is proved to be involved in tumor progression and treatment. Immune cells are most likely to be affected by TME [7]. Among them, tumor-infiltrating cells have attracted a lot of attention because of their duality and importance. They can target tumor cells and show anti-tumor activity. On the contrary, they can also exhibit pro-tumor activity and promote tumor development and metastases. In addition, regulatory T cells (Tregs) are considered to secrete suppressive cytokines such as TGF-β and IL-10, express cytotoxic T lymphocyte–associated protein 4 (CTLA-4), and significantly correlate with tumor progression in HNSCC [8]. Therefore, the investigation of TME in HNSCC to reveal the underlying mechanisms is important for the improvement of the diagnosis and treatment of HNSCC.

In the present study, we used weight gene co-expression network analysis (WGCNA) to identify immune infiltration-related gene modules in HNSCC and constructed a prognostic model based on LASSO Cox regression analysis. Nine genes in our risk model significantly influenced patients’ survival, and were effectively validated in the expression levels of mRNA and protein using GEPIA, HPA database and immunohistochemical method. We further investigated the landscape of immune infiltration, immunotherapy sensitivity and tumor mutation in two risk groups. Our results might help us deeply understand how TME affects patient’s clinical outcome and offer novel prognostic and therapeutic target of HNSCC.

## Methods

### Dataset acquisition and preparation

The RSEM normalized RNA-seq data of the TCGA HNSCC cohort was retrieved from the Broad GDAC firehose (http://gdac.broadinstitute.org/). The clinical phenotype of HNSCC patients was obtained from the UCSC Xena (https://xenabrowser.net/). Data with incomplete clinical information, overall survival less than 30 days and outliers identified by clustering algorithm were deprecated. Total 491 qualified HNSCC patients were included in our study. The validation datasets (GSE65858, GSE41613, GSE686) and cohort treated with immunotherapy targeting PD-1/PD-L1 (GSE159067) were retrieved from GEO (https://www.ncbi.nlm.nih.gov/geo/), and underwent the same preparation procedures. The expression array of GSE65858 was based on GPL10558 (Illumina HumanHT-12 V4.0 expression beadchip) and included 267 qualified HNSCC patients. The expression array of GSE41613 was based on GPL570 (Affymetrix Human Genome U133 Plus 2.0 Array) and included 97 qualified HNSCC patients. The expression array of GSE686 was based on GPL503 (Agilent Human 1 cDNA Microarray) and included 71 qualified HNSCC patients. The relevant clinical characteristics was presented in Table 1.

**Table 1:** Clinical characteristics of HNSCC patients in TCGA and validation dataset

Variables	TCGA-HNSCC (***n*** = 491)	GSE65858 (***n*** = 267)	GSE41613 (***n*** = 97)	GSE686 (***n*** = 71)
**Age**				
≤ 60	244 (49.7%)	156 (58.4%)	50(51.55%)	49 (69.01%)
> 60	247 (50.3%)	111 (41.6%)	47 (48.45%)	22 (30.99%)
**Gender**				
Female	131 (26.7%)	47 (17.6%)	31 (31.96%)	8 (11.27%)
Male	360 (73.3%)	220 (82.4%)	66 (68.04%)	63 (88.73%)
**Vital status**				
Alive	280 (57.0%)	176 (65.9%)	46 (47.42%)	53 (74.65%)
Dead	211 (43.0%)	91 (34.1%)	51 (52.58%)	18 (25.35%)
**Histologic grade**				
G1	54 (11.0%)	NA	NA	NA
G2	293 (59.7%)	NA	NA	NA
G3	120 (24.4%)	NA	NA	NA
G4	7 (1.4%)	NA	NA	NA
GX	17 (3.5%)	NA	NA	NA
**Stage**				
I	20 (4.1%)	17 (6.3%)	41 (42.27%)	0
II	96 (19.5%)	37 (13.9%)		12 (16.90%)
III	103 (21.0%)	37 (13.9%)	56 (57.73%)	18 (25.35%)
IV	272 (55.4%)	176 (65.9%)		41 (57.75%)
**T classification**				
T1	34 (6.9%)	34 (12.7%)	NA	NA
T2	147 (29.9%)	80 (30.0%)	NA	NA
T3	132 (26.9%)	57 (21.3%)	NA	NA
T4	178 (36.3%)	96 (36.0%)	NA	NA
**N classification**				
N0	240 (48.9%)	93 (34.8%)	NA	NA
N1	79 (16.1%)	32 (12.0%)	NA	NA
N2	155 (31.6%)	130 (48.7%)	NA	NA
N3	9 (1.8%)	12 (4.5%)	NA	NA
NX	8 (1.6%)	0	NA	NA
**M classification**				
M0	475 (96.7%)	261 (97.8%)	NA	NA
M1	6 (1.2%)	6 (2.2%)	NA	NA
MX	10 (2.0%)	0	NA	NA

**Fig. 1:**
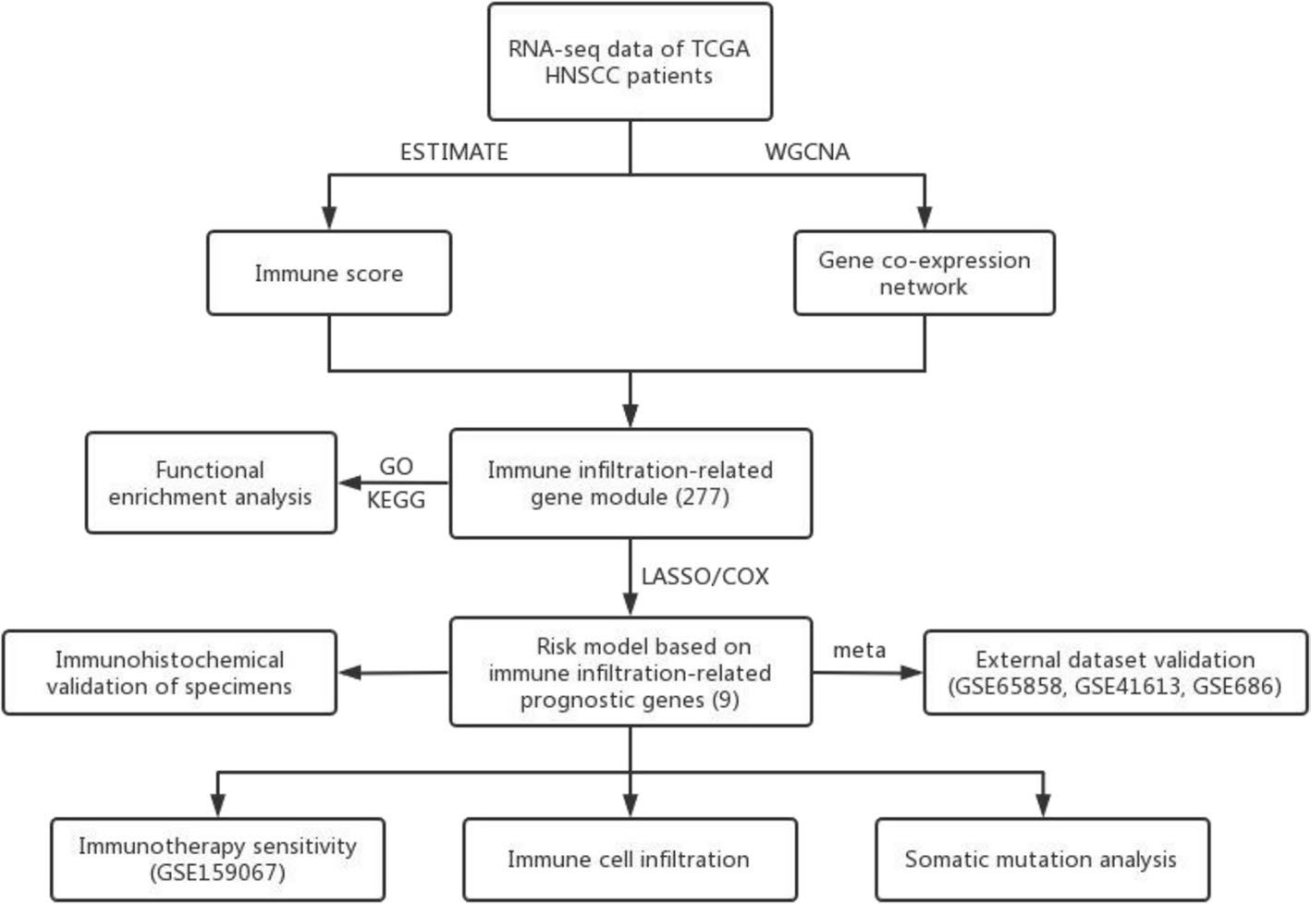
Basic flow chart of this study

The dataset used in this study was public and open access and all procedures followed the data access policies and publication guides of the database. For the study on public data, no approval or informed consent by local ethics committee was required. The complete procedures used in this study are displayed as a flow chart in Fig. 1.

### Investigation of the association between tumor microenvironment and prognosis

To evaluate the tumor microenvironment of HNSCC patients, the Estimation of Stromal and Immune cells in Malignant Tumors using Expression data (ESTIMATE) was used to calculate the immune score and ESTIMATE score for each sample via R package “estimate” [9]. According to these scores, HNSCC patients were divided into the high group (score > 75 percentile) and the low group (score < 25 percentile). R package “survminer” was used to plot Kaplan-Meier survival curves for the groups of different scores.

### Gene set enrichment analysis (GSEA) and gene set variation analysis (GSVA)

To discover the differences in immune-related, tumor-related and signaling pathways between HNSCC samples and normal samples, we performed GSEA and GSVA analysis on HNSCC samples and normal samples from TCGA using the R software packages “clusterprofiler” and “GSVA” respectively. The gene sets for these functions and pathways were obtained from the GSEA website (https://www.gsea-msigdb.org/gsea/index.jsp).

### Construction of weighted gene co-expression network

The weighted gene co-expression network analysis (WGCNA) was used to build gene co-expression network via R package “WGCNA” [10]. The RSEM normalized matrix with top 5000 variable genes was input. First, the absolute value of the correlation coefficient between every two genes was calculated to build a gene expression similarity matrix, which was then transformed into an adjacency matrix. The optimal soft thresholding β was selected to ensure scale independence over 0.90 for the construction of a scale-free co-expression network. Next, the adjacency matrix was converted in a topological overlap matrix (TOM) to store the connectivity between genes. Finally, hierarchical clustering and the method of dynamic cut tree was applied to identify co-expression gene modules. Significant gene modules positively correlated with immune score were defined as immune infiltration-related gene modules. The module-trait relationship analysis calculated the module member (MM) and gene significance (GS) to evaluate the correlation between specific gene modules and phenotypes.

### Functional enrichment analysis of immune infiltration-related gene modules

Gene Ontology (GO) and Kyoto Encyclopedia of Genes and Genomes (KEGG) pathway enrichment of the intriguing modules from WGCNA were performed using R package “clusterProfifiler”. Enriched terms with adjusted *P* value < 0.05 and gene counts ≥3 were considered significant. The Benjamini and Hochberg method was used to adjust P value.

### Nine-gene model of HNSCC based on immune infiltration-related prognostic genes

We used univariate, the least absolute shrinkage and selection operator (LASSO), and multivariate cox regression to filter significant prognostic genes from immune infiltration-related gene modules. First, univariate cox regression was applied to identify genes significantly correlated with overall survival (*P* < 0.05). LASSO is a linear regression algorithm using shrinkage for survival analysis [11]. LASSO cox regression further narrowed the number of immune infiltration-related prognostic genes in HNSCC cohort. Ten-fold cross validation was performed to minimize the instability of the results and the optimal parameter lambda was selected based on 1-SE (standard error). Then multiple cox regression was used to evaluate the independence of reserved genes from LASSO. Considering the prediction performance and variable independence in the meantime, a nine-gene signature model was finally established. The risk score of each patient was calculated as the coefficients from multivariate cox regression of genes multiplied by their expression levels. Nomogram is an analogue tool to combine complex and multiple variables into a simple chart for the prediction of overall survival [12]. In our study, basic clinical features and the risk score from the nine-gene signature model were included to build a nomogram which can predict the survival probability at 1 year, 3 years, and 5 years. The ROC analysis was also used to access the sensitivity and specificity of the nomogram.

### Comparison of expression differences and survival analysis for model candidate genes

Gene expression profiling interactive analysis (GEPIA, http://gepia.cancer-pku.cn/) was used to visualize the expression level of model candidate genes in tumor and normal samples from TCGA HNSCC dataset. Differentially expressed genes were identified under the criterion of logFC > 0.5 and *P* value < 0.01. The Kaplan-Meier survival curves for each model candidate gene were plot by R package “survminer”.

### Meta-analysis of the nine-gene prognostic model

A meta-analysis was performed using the “meta” R package and 4 HNSCC datasets from TCGA and GEO were included. Heterogeneity among the datasets was assessed using the Chi^2^ and the I^2^ statistic. *p*-values < 0.05 were considered statistically significant.

### Immunohistochemical validation of clinical specimens

To validate the different expression of model candidate genes at protein level, clinical specimens of HNSCC patients and the human protein atlas (HPA, http://www.proteinatlas.org) were used for immunohistochemical analysis. Tumor tissues were collected from 8 HNSCC patients diagnosed in the Third Xiangya Hospital from January 2020 to December 2020. These tissues were formalin-fixed and paraffin-embedded. This study was approved by the Ethics Committee of the Third Xiangya Hospital (No. 21158), and the study was in accordance with the principle of the Helsinki Declaration II. Immunohistochemical procedures were performed as previously described [13]. The following antibodies were used in our immunohistochemistry experiments: Anti-ISG20 antibody (1:300 dilution; ab135842; Abcam Biochemicals); Anti- CTSL antibody (1:200 dilution; 10,938–1-AP; Proteintech). IHC results for ISG20 and CTSL were assessed by ImageJ software, optical density (OD) was measured, and immune response scores were assessed with the IHC Profiler plugin. The IHC Profiler uses the average gray value (staining intensity) and the percentage of positive area (staining area) of positive cells as the indicators of IHC, and finally obtains four scores: High positive (3+), Positive (2+), Low Positive (1+) and Negative (0) [14]. The Human Protein Atlas (HPA, https://www.ptrotinatlas.org/) provided us with immunohistochemical data for TBC1D2, WIPF1, TMEM173 and C5orf15 in HNSCC and normal tissues. The degree of staining is divided into four levels: high, medium, low, and not detected. All methods were carried out in accordance with relevant guidelines and regulations.

### Prediction of immunotherapy sensitivity of HNSCC patients

The filtered immunotherapy-treated cohort, GSE159067 included 101 HNSCC patients receiving PD-1/PD-L1 inhibitors, whose treatment outcomes were divided into progressive disease (PD), stable disease (SD), partial response (PR), and complete response (CR). The risk scores and groups of each patient were calculated for further statical comparisons. Through the online platform TIDE (http://tide.dfci.harvard.edu), we used the Tumor Immune Dysfunction and Exclusion (TIDE) algorithm to predict the response of TCGA-HNSCC patients to immune checkpoint blockade (ICB) therapy, and investigated their correlation with the risk score of the immune infiltration-related gene prognostic model [15].

### Landscape of immune cell infiltration in HNSCC

CIBERSORT were applied to evaluate the characteristics of immune cell infiltration in TCGA HNSCC cohort, so as to seek for the potential association with risk groups and model genes. CIBERSORT calculates immune cell composition in each HNSCC patient based on a deconvolution algorithm [16]. The correlation analysis of immune cell types and risk score model was performed by R package “corrplot”.

### Somatic mutation analysis of HNSCC patients

The genomic mutation data were retrieved from R package “TCGAmutations” and visualized by the functions of R package “maftools”. The tumor mutation burden (TMB) scores of HNSCC patients were downloaded from a published study [17].

### Statistical analysis

Most analyses were conducted in R version 4.0.5 (https://www.r-project.org/) and online analytical websites. Kruskal-Wallis nonparametric test was used to judge the statistic difference between more than two groups under undetermined variances, and Wilcoxon rank sum test was used for pairwise comparisons. In two-group comparison, t test was only applied to continuous variables with normal distribution and equal variance. The overall survival of risk groups was compared using log-rank test. The significance level was set as 0.05.

## Results

### Association between immune microenvironment and tumor progression in HNSCC

**Fig. 2:**
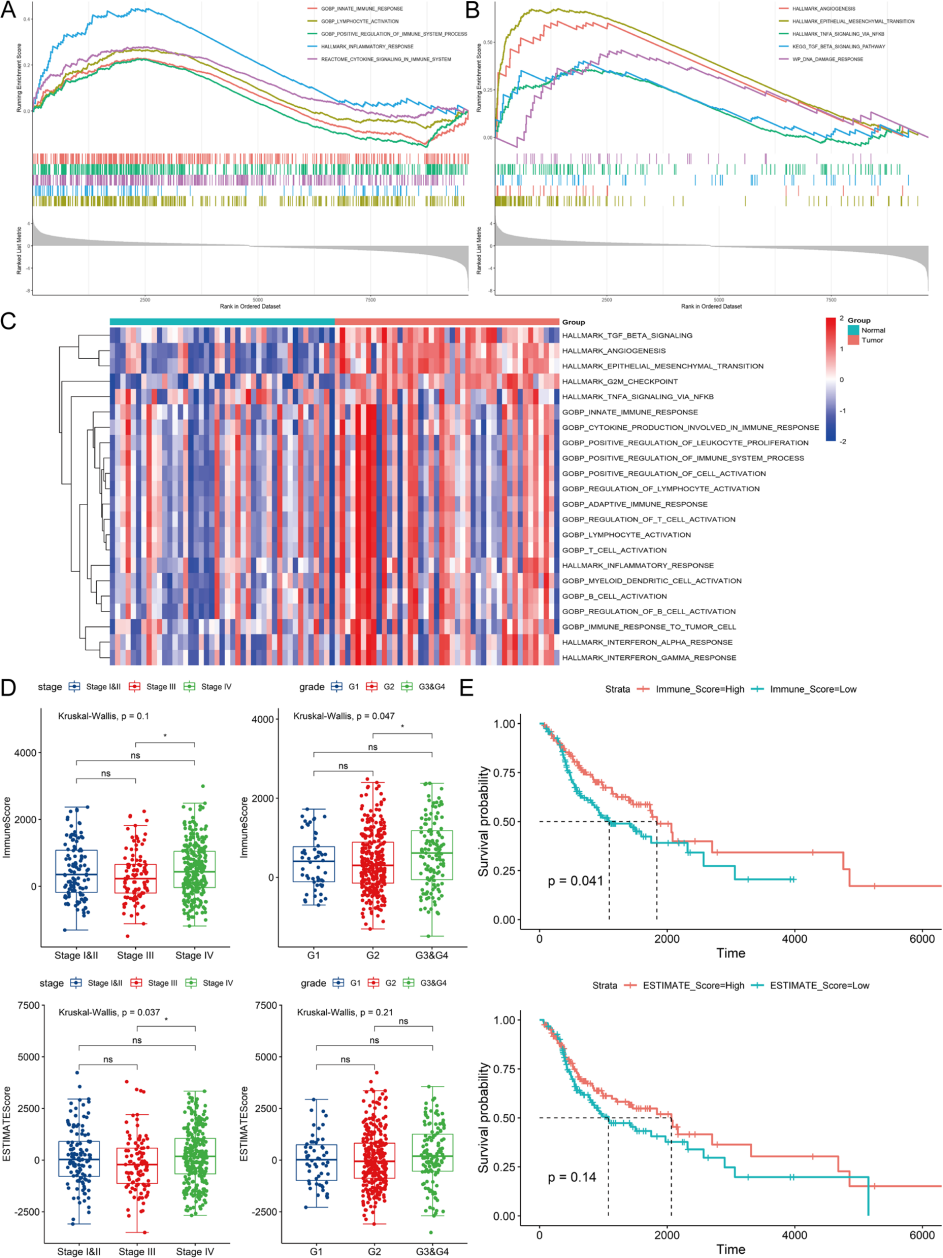
The landscape of immune infiltration microenvironment delineated by GSEA, GSVA and ESTIMATE analysis in HNSCC. **A**, **B** The GSEA results of significant pathways related to immune response and tumor microenvironment. **C** The heatmap of GSVA scores of biological processes involving immune response and tumor microenvironment in HNSCC and adjacent tissues. **D** The boxplot and statistical comparisons of immune and ESTIMATE scores of different tumor stages or grades. **E** The survival curves based on immune and ESTIMATE scores

Biological processes related to immune microenvironment and tumor progression were enriched in TCGA-HNSCC cohort according to GSEA (Fig. 2A-B). The results of GSVA between HNSCC and adjacent normal tissues showed prominent activation of immune- and tumor-related pathways (Fig. 2C). The ESTIMATE algorithm assigned scores of tumor microenvironment to each patient based on their expression profiles, and the immune score and ESTIMATE score were statistically compared between tumor stages and grades. As shown in Fig. 2D, the immune score was significantly correlated with histological grade (*p* = 0.047) and the ESTIMATE score was correlated with tumor stage (*p* = 0.037). The immune score in G3 & G4 was significantly higher than that in G2 (mean 590.37 (SD 849.84) vs. mean 386.80 (SD 751.48), *p* < 0.05). Besides, the ESTIMATE score in Stage IV was significantly higher than that in Stage III (mean 192.72 (SD 1336.30) vs. mean 181.70 (SD 1328.08), *p* < 0.05). Furthermore, Kaplan-Meier survival curves of patient groups based on several scores revealed better survival in patients with lower immune scores (Fig. 2E, log-rank *p* = 0.041).

### Identification of immune infiltration-related gene modules by WGCNA

**Fig. 3:**
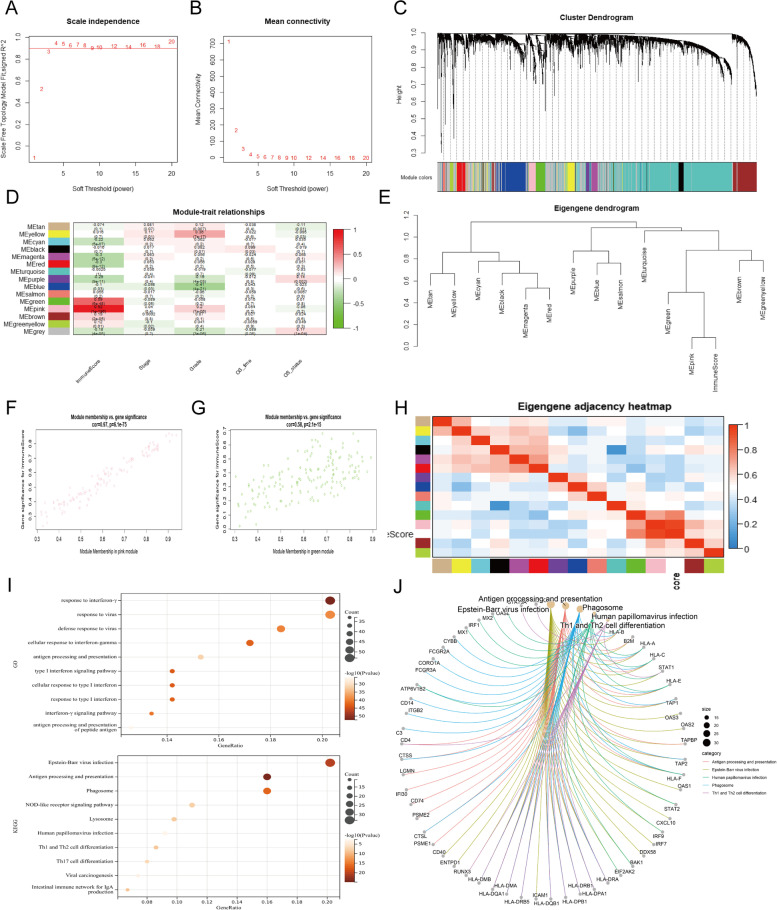
Identification of immune infiltration-related gene modules by WGCNA. **A**, **B** Screening of the most suitable soft threshold to build a scale-free network with ideal scale independence and mean connectivity. **C** Clustering dendrogram of co-expression gene modules. **D** The correlation between modules and traits. The correlation coefficient and *p*-value are presented in each cell. **E** Eigengene dendrogram of gene modules and immune score. **F**, **G** Correlation scatter plots of module membership and gene significance in immune infiltration-related gene modules. **H** The eigengene adjacency heatmap of gene modules and immune score colored by white. **I** Functional enrichment of immune infiltration-related gene modules by GO and KEGG analysis. The top 10 biological processes and pathways are displayed. **J** Cnetplot of enrichment pathways and annotated genes

Since immune scores were associated with patients’ survival in HNSCC, we investigated related co-expression genes using the WGCNA algorithm. The optimal soft threshold β was selected to achieve ideal scale independence and mean connectivity before constructing the weighted network with efficiency (Fig. 3A-B). Clustering dendrogram was calculated to generate co-expression gene modules (Fig. 3C). Based on the correlation between gene modules and the immune score in module-trait relationship heatmap, pink and green modules were considered as immune infiltration-related gene modules and enrolled in further analysis (Fig. 3D). The eigengene dendrogram indicated the most significant correlation between immune score and the two gene modules (Fig. 3E). Both modules exhibited significant module membership relevance to gene significance (Fig. 3F-G, cor = 0.97, *p* = 6.1e− 75 for the pink module; cor = 0.58, *p* = 2.1e− 15 for the green module). The adjacency heatmap also supported the high correlation between the two modules and the immune score (Fig. 3H). Total 277 genes in the pink and green modules were then extracted for functional enrichment analysis. The GO enrichment of biological processes was focused on response to interferon-gamma, type I interferon and virus (Fig. 3I). Results of KEGG enrichment showed that immune infiltration-related genes were significantly associated with diseases and pathways including: EB virus infection, antigen processing and presentation, phagosome, human papillomavirus infection, Th1 and Th2 cell differentiation (Fig. 3I-J).

### Construction of a nine-gene prognostic model for survival prediction of HNSCC

**Fig. 4:**
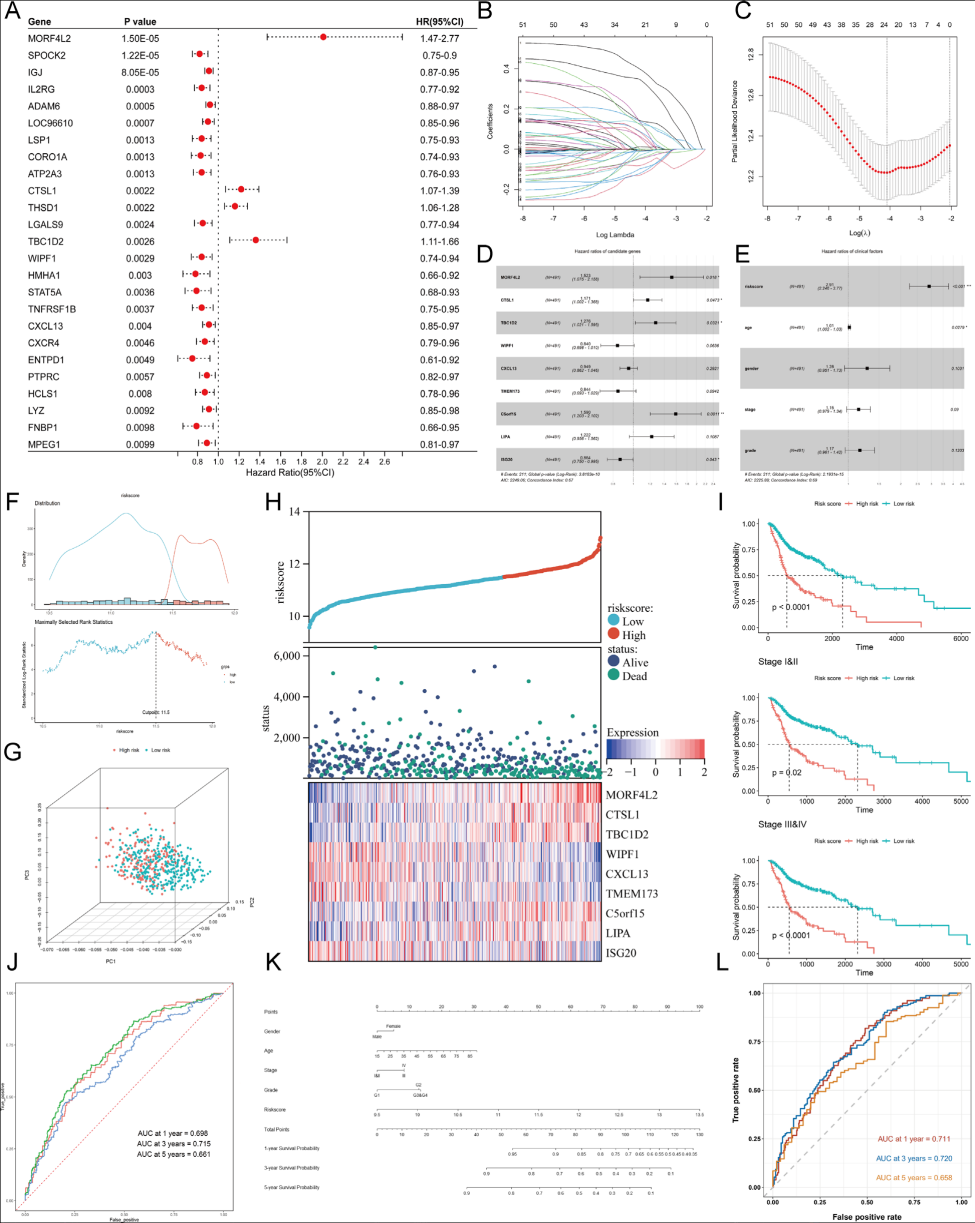
Identification of nine immune infiltration-related prognostic genes to build a risk score model. **A** Forest plot of prognostic module genes with *P* < 0.01 by univariate Cox regression. **B**, **C** Ten-time cross-validation of the LASSO model and coefficient profile of filtered prognostic genes. **D** Construction of the prognostic model by multivariate Cox regression. The hazard ratios (HRs) and 95% confidence intervals (CIs) of each candidate gene are shown. **E** The multivariate cox regression of the association between clinical factors (including the risk score) and survival. **F** The optimal cut point selected by the maximum standard log-rank statistics in HNSCC cohort. **G** PCA based on 277 immune infiltration-related genes showing different immune phenotypes in two risk groups. **H** The model gene expression heatmap combined with the distribution of risk scores and the survival of patients in two risk groups. **I** Kaplan-Meier survival curves of two risk groups in the whole and stage-divided HNSCC cohorts. **J** ROC curves based on risk score in HNSCC cohort within 1–5 years. **K** Nomogram combining risk score with clinical information. **L** ROC curves evaluating the predictive efficacy of the nomogram for the overall survival within 1–5 years

To screen prognostic immune infiltration-related genes, cox regression analysis was applied for genes in the pink and green modules. First, univariate cox regression identified 52 significant prognostic genes and genes with *p* < 0.01 were visualized by forest plot (Fig. 4A). LASSO cox regression was used to filter the most stable prognostic genes with optimal parameter lambda, which ensured that the sum of LASSO regression coefficients was below a fixed threshold. Cross validation was performed to prevent the model from over-fitting (Fig. 4B-C). Considering the prognostic effect of the entire model and the independence of members, nine genes were finally reserved to build an immune infiltration-related gene prognostic model. The hazard ratios of candidate genes calculated by multivariate cox regression were shown in Fig. 4D. In these model genes, MORF4L2, CTSL1, TBC1D2, C5orf15, and LIPA were risk genes (HR > 1), while WIPF1, CXCL13, TMEM173, and ISG20 were associated with low risk (HR < 1). Based on the coefficients derived from the multivariate cox regression and the expression levels of nine candidate genes, risk scores were estimated for each patient: risk score = 0.42049 × expression of MORF4L2 + 0.15774 × expression of CTSL1 + 0.24388 × expression of TBC1D2 + − 0.17454 × expression of WIPF1 + − 0.05195 × expression of CXCL13 + − 0.16907 × expression of TMEM173 + 0.46373 × expression of C5orf15 + 0.20081 × expression of LIPA + − 0.14594 × expression of ISG20. Furthermore, the multivariate cox regression of clinical factors demonstrated that the risk score was an independent prognostic factor for HNSCC (Fig. 4E, *p* < 0.001).

On the basis of risk scores calculated before, 491 HNSCC patients in TCGA were divided into high-risk group and low-risk group via the maximally selected rank method (Fig. 4F). The expression profiles of immune infiltration-related gene modules and model candidate genes through PCA indicated distinct immune phenotypes in risk groups (Fig. 4G). Besides, the model gene expression heatmap along with the distribution of risk scores and survival status in two patient groups was shown in Fig. 4H. Kaplan-Meier survival curves showed a significant difference between two risk groups, and the prognosis of patients in the high-risk group was significantly worse (Fig. 4I, log-rank *p* < 0.05). ROC curves with 1-, 3-, and 5-year AUC were also plotted to evaluate the prediction efficacy of the risk model. The AUCs corresponding to 1, 3, and 5 years of survival were 0.698, 0.715, 0.661, respectively, which suggested high sensitivity and specificity of the nine-gene prognostic model (Fig. 4J). To enhance the clinical usability of the nine-gene prognostic model, we developed a nomogram with five independent factors including gender, age, TNM stage, histological grade, and risk score in TCGA cohort (Fig. 4K). The ROC curve showed that the 1-, 3-, and 5-year AUCs were 0.711, 0.720, 0.658, respectively, which represented a better predictive ability compared with risk score (Fig. 4L).

### Expression difference and prognostic effect of model candidate genes

**Fig. 5:**
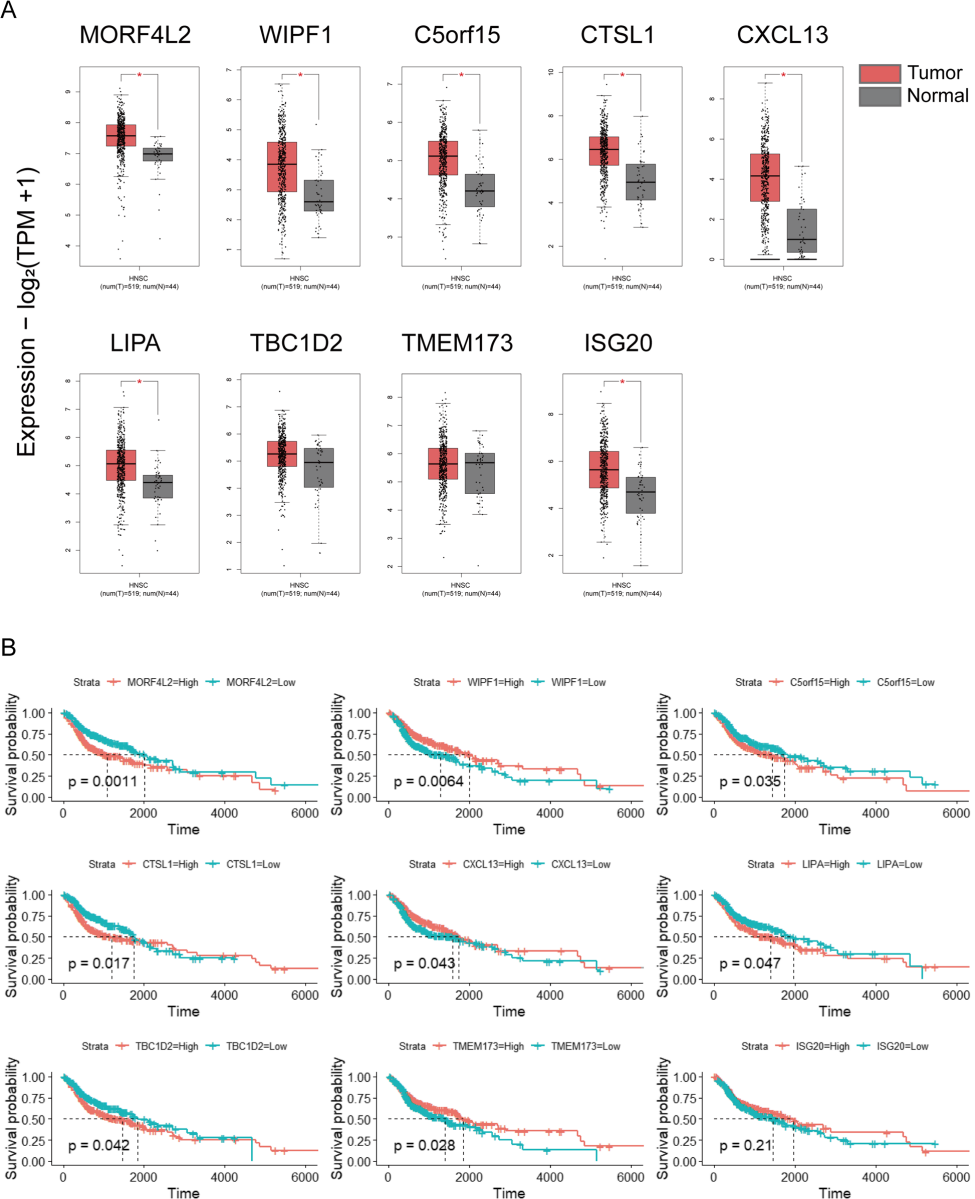
Investigation of the expression difference and prognostic effect of 9 candidate genes in TCGA HNSCC cohort. **A** The mRNA expression levels of 9 candidate genes in tumor and normal samples. **B** Kaplan-Meier survival curves for 9 candidate genes

We used GEPIA, an analytical website of TCGA database, to investigate the dynamic expression changes of nine model genes in tumor and adjacent normal tissues. Results showed that the expression level of genes including MORF4L2, CTSL1, WIPF1, CXCL13, C5orf15, LIPA, and ISG20 significantly elevated in tumor tissues (Fig. 5A). Moreover, Kaplan-Meier survival curves were also drawn for candidate genes, respectively. HNSCC patients with high expression levels of MORF4L2, CTSL1, TBC1D2, C5orf15, LIPA and low expression levels of WIPF1, CXCL13, TMEM173 had poor outcomes (Fig. 5B; log-rank *p* < 0.05).

### Meta-analysis of the nine-gene prognostic model

**Fig. 6:**
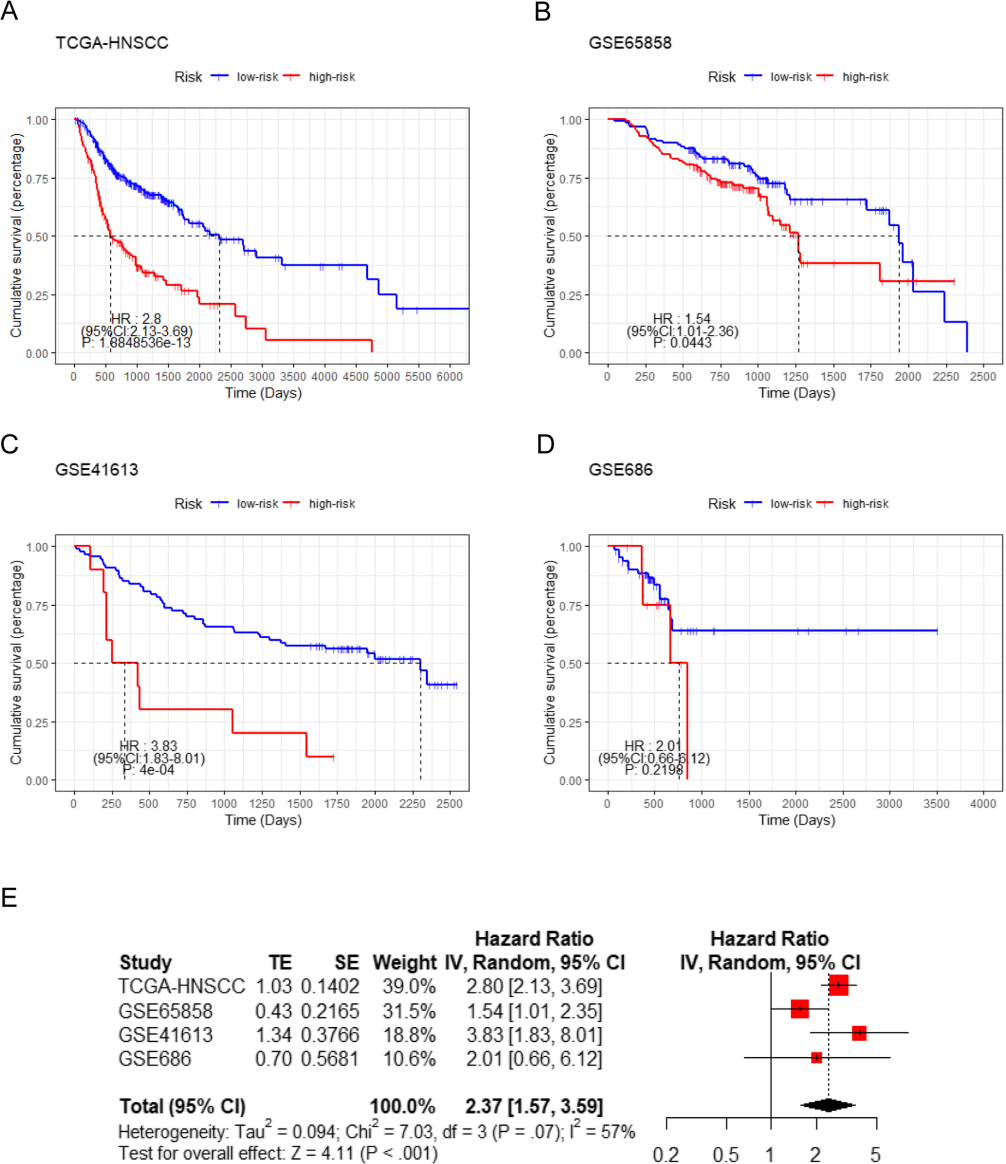
Meta-analysis of the nine-gene signature model. **A** The Kaplan-Meier survival curve and hazard ratio (HR) based on TCGA-HNSCC. **B** The Kaplan-Meier survival curve and HR based on GSE65858. **C** The Kaplan-Meier survival curve and HR based on GSE41613. **D** The Kaplan-Meier survival curve and HR based on GSE686. **E** Meta-analysis of survival data for the nine-gene signature model. TE: estimate of treatment effect; SE: standard error; HR: hazard ratio; CI: confidence interval

Through the previously constructed nine-gene prognostic model, we risk-scored the cases in the HNSCC datasets included in the meta-analysis, and obtained their Kaplan-Meier survival curve and hazard ratio(HR)(Fig. 6A-D). The meta-analysis based on these results confirmed that the nine-gene prognostic model risk score was associated with prognosis in HNSCC (HR = 2.37, 95% CI: 1.57–3.59, Fig. 6E).

### Immunohistochemical validation of the protein expression of nine candidate genes

**Fig. 7:**
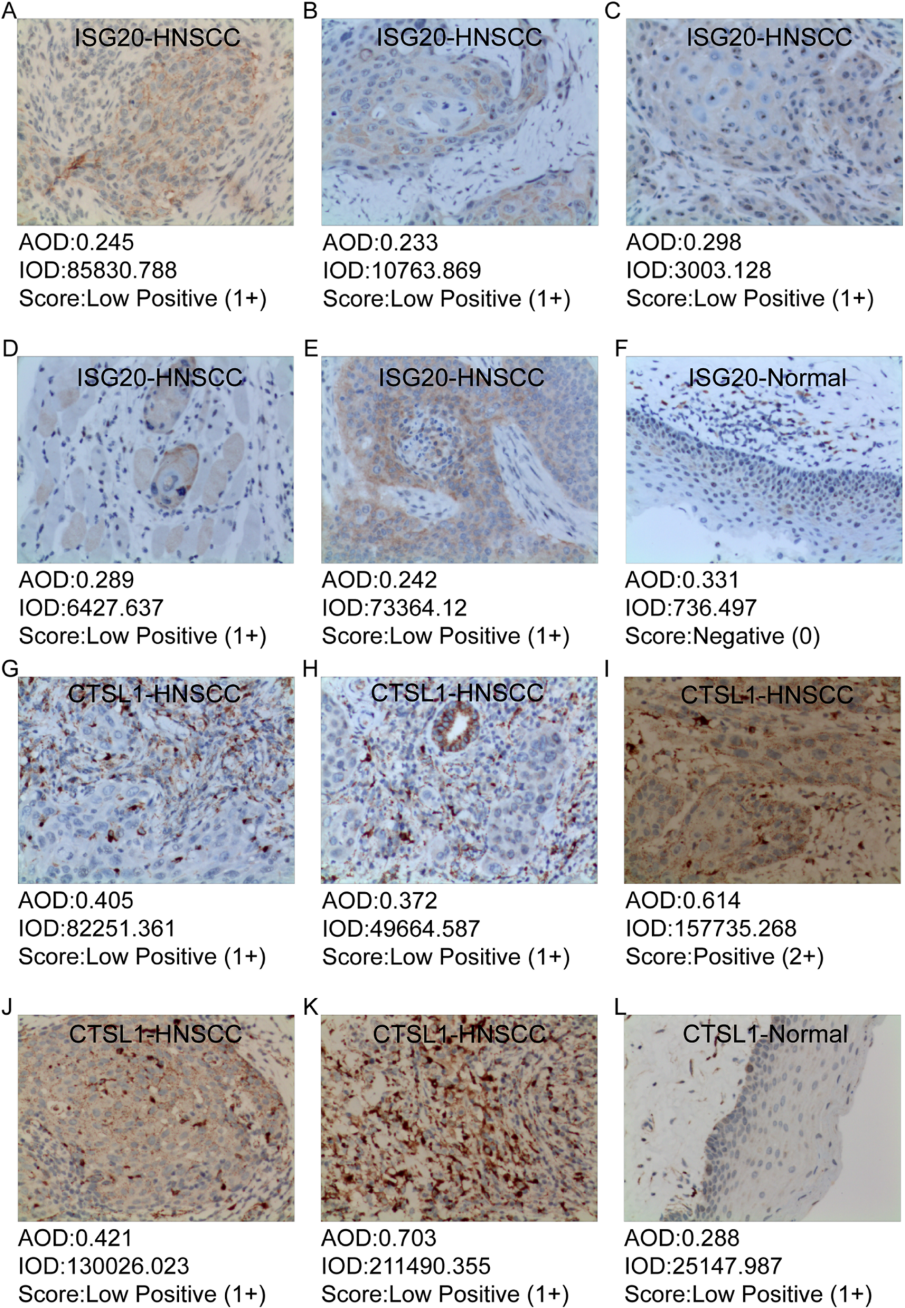
Immunohistochemistry analysis of the protein expression of ISG20 and CTSL1 in HNSCC and normal tissues. **A**-**E** The expression of ISG20 was detected by immunohistochemistry in 5 patients with HNSCC (Magnification × 200). **F** The expression of ISG20 was detected by immunohistochemistry in normal head and neck squamous cell tissue (Magnification × 200). **G**-**K** The expression of CTSL1 was detected by immunohistochemistry in 5 patients with HNSCC (Magnification × 200). **L** The expression of CTSL1 was detected by immunohistochemistry in normal head and neck squamous cell tissue (Magnification × 200). AOD: average optical density; IOD: integral optical density

**Fig. 8:**
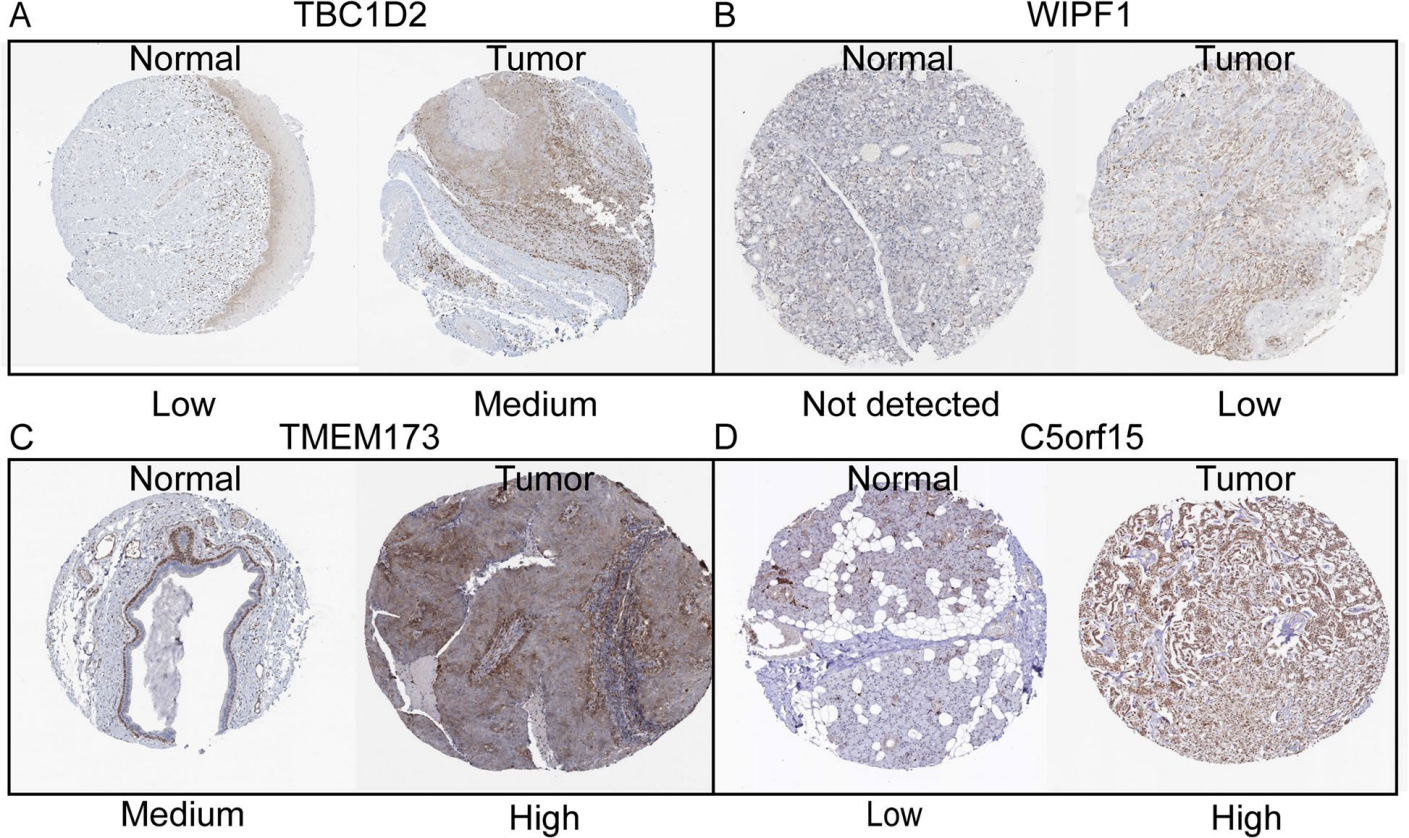
Immunohistochemistry of several prognostic signatures based on the HPA. **A** Protein expression levels of TBC1D2 in HNSCC and normal tissue. **B** Protein expression levels of WIPF1 in HNSCC and normal tissue. **C** Protein expression levels of TMEM173 in HNSCC and normal tissue. **D** Protein expression levels of C5orf15 in HNSCC and normal tissue

We further validated the difference of protein expression levels for most genes in the risk model. To verify the difference in the protein expression of ISG20 and CTSL1 between HNSCC tissues and normal squamous epithelium of the head and neck, we performed Immunohistochemistry analysis on HNSCC paraffin sections. Immunohistochemistry analysis suggested that the expression levels of ISG20 and CTSL1 were significantly higher in HNSCC tissue quantified by the antibodies ab135842 (Fig. 7A-F) and 10,938–1-AP (Fig. 7G-L). Based on the results of immunohistochemistry from HPA, we compared the protein expression of TBC1D2, WIPF1, TMEM173 and C5orf15 in HNSCC tissues and normal squamous epithelium typically located in the head and neck. According to the HPA results, the protein expression levels of these genes were significantly different between HNSCC tissues and normal tissues (Fig. 8A-D). Genes including MORF4L2, CXCL13, and LIPA showed minor difference of staining intensity in HPA.

### Immunotherapy sensitivity and immune infiltration of nine-gene prognostic model

**Fig. 9:**
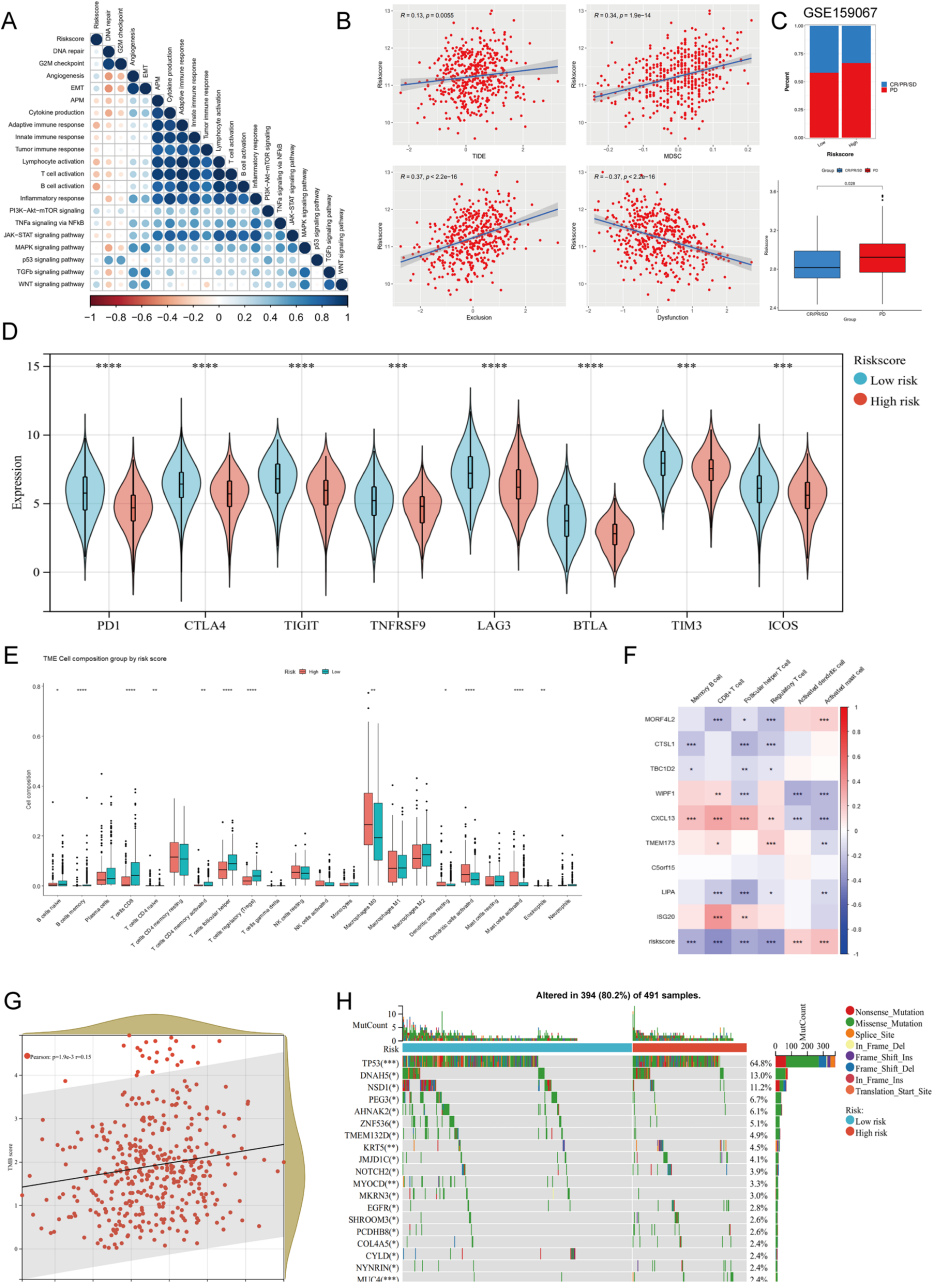
The profiles of immunotherapy sensitivity, immune infiltration and somatic mutation in HNSCC patients. **A** The correlation between the risk score and known biological processes and signaling pathways in tumor microenvironment. **B** The correlation between risk scores and tumor immune dysfunction and exclusion (TIDE) scores, myeloid-derived suppressor cell (MDSC), T cell exclusion scores, and T cell dysfunction scores. **C** The proportion of patients with different immunotherapy responses in risk groups from dataset GSE159067 and the difference of risk scores between immunotherapy response groups. **D** The expression levels of immune checkpoint genes in two risk groups. **E** The calculated proportion of 22 immune cells in two risk groups. **F** Heatmap showing the correlation significance between immune cells and model candidate genes. **G** The correlation between the risk score and tumor mutation burden (TMB) score. **H** The mutation landscape of HNSCC patients in two risk groups. The barplot represents the composition of mutation type and the percentage represents the mutation frequency of each gene. PD: progressive disease; SD: stable disease; PR: partial response; CR: complete response. * *p* < 0.05; ** *p* < 0.01; *** *p* < 0.001; **** *p* < 0.0001

The GSVA scores of biological processes and signaling pathways were calculated for each patient to investigate their correlation with the risk score. It was suggested that the risk score was positively associated with EMT, angiogenesis, PI3K-Akt-mTOR signaling and WNT signaling pathway, but was negatively associated with immune responses and immune cell activation (Fig. 9A). TIDE algorithm provided us with a quantitative metric of immunotherapy responsiveness. Higher TIDE scores indicated weaker responses. Results showed the risk score was positively correlated with TIDE score (R = 0.13, *p* = 0.0055), which speculated better responses of immunotherapy in HNSCC patients with lower risk scores (Fig. 9B). The risk score was also positively correlated with myeloid-derived suppressor cell (MDSC) and T cell exclusion, while negatively correlated with T cell dysfunction. In the immunotherapy-treated cohort, the proportion of PD patients in high-risk group was elevated and risk scores in PD group were significantly higher than that in CR/PR/SD group (Fig. 9C, *p* < 0.05). Immune checkpoint genes including PD1, CTLA4, TIGIT, TNFRSF9, LAG3, BTLA, TIM3, ICOS were highly expressed in the low-risk group, which supported that low-risk patients based on the nine-gene prognostic model might benefit more from immunotherapy (Fig. 9D). Using CIBERSORT to calculate the proportion of 22 immune celltypes in HNSCC patients, we found that over half of immune celltypes significantly altered in different risk groups (Fig. 9E). Among them, six specific immune celltypes showed apparent distinction (*p* < 0.0001): memory B cells, CD8+ T cells, follicular helper T cells, and regulatory T cells significantly decreased in the high-risk group, while activated dendritic cells and activated mast cells significantly increased. Further investigation showed that most model candidate genes except C5orf15 were significantly correlated with risk-related immune celltypes to some degree (Fig. 9F). Notably, CXCL13 was significantly correlated with all six immune cells. On the other hand, CD8 + T cell, Follicular T cells, and regulatory T cells had the most significant correlation with model candidate genes. Moreover, the tumor mutation burden (TMB) scores were positively associated with risk scores in HNSCC patients (Fig. 9G). We also estimated the incidence of somatic mutations in two risk groups using genomic data. Results revealed that TP53 gene exhibited the highest mutation frequency followed by DNAH5. In addition, their mutation frequency was significantly higher in high-risk patients (Fig. 9H, *p* < 0.05).

## Discussion

In recent years, the tumor microenvironment has been regarded as a pivotal role in the progression of cancers including HNSCC [18]. The immune cells, stromal cells and extracellular components closely interact with tumor cells and form a complicated regulatory network to influence tumor growth and metastasis. The tumor often induces a suppressive microenvironment via impairing the function of both innate and adaptive immune cells to escape host’s immune surveillance [19]. Despite the application of traditional immune therapies in HNSCC, a large portion of patients show limited or no responses to current drugs. It is urgent and inevitable to find novel immune infiltration-related molecular targets in HNSCC tumor microenvironment.

Bioinformatic analysis has been widely used to investigate the tumor microenvironment profiles in various cancers. A recent study focusing on immune microenvironment of clear cell renal cell carcinoma identified critical immune subgroups via unsupervised consensus clustering and filtered hub genes from WGCNA modules [20]. Clustering procedures for modeling were applied in another study on the prognosis and immunotherapy response of lung squamous cell carcinoma [21]. LASSO regression analysis and multivariate Cox proportional model were selected as robust methods for the construction of prognostic gene signature [20, 22].

In our study, immune scores were calculated to estimate the infiltrating level of immune cells by ESTIMATE algorithm which was widely used to infer tumor purity. Compared with other immune infiltration-related HNSCC risk prediction models, our model not only has good predictive performance on prognosis, but also can predict patient response to immunotherapy. Unlike other validation methods using single or multiple datasets, we used a prognostic meta-analysis to test the applicability and stability of our model.

Our results suggested that patients with high immune scores had significantly ameliorated prognosis. We propose the hypothesis that enhanced immune infiltration levels in HNSCC promote anti-tumor responses and thus contain the tumor progression. On the contrary, low immune score indicating suppressed immune response can be a risk factor for the prognosis of HNSCC patients. Consistent with our findings, high immune score was significantly correlated with favorable survivals in gastric cancer and osteosarcoma [23, 24]. But elevated immune score can also indicate poor overall survivals as described in clear cell renal cell carcinoma [25]. It is speculated that the practical effect of immune infiltration on tumor microenvironment is attributed to not only the quantity of infiltrated immune cells, but also the functional activity and interactive patterns with the tumor.

The risk model we constructed consists of nine genes: MORF4L2, CTSL1, TBC1D2, WIPF1, CXCL13, TMEM173, C5orf15, LIPA, and ISG20. MORF4L2 is a component of the NuA4 histone acetyltransferase complex involved in the activation of oncogene and proto-oncogene-mediated growth induction, and replicative senescence, apoptosis, and DNA repair. Cathepsin L (CTSL1), a lysosomal cysteine protease member, is mainly involved in the terminal degradation of intracellular phosphorylated proteins [26]. Increasing evidences indicate that CTSL1 is highly and specifically expressed in various cancers [27, 28]. TBC1D2 is a GTPase-activating protein of Rab7 GTPase. In breast cancer cells, persistent Rac1 activity enhanced escape of β4 integrin from lysosomal degradation depending on actin-related protein 2/3 and TBC1D2 [29]. WIPF1, also known as the WASP-interacting protein (WIP), drives the oncogenic activity of mutant p53. Knockdown of WIPF1 in glioblastoma and breast cancer cells expressing mutant p53 reduced the proliferation and growth ability of cancer stem-like cells and decreased the expression of cancer stem-like markers such as CD44, CD133, and TAZ/YAP. WIPF1 knockdown inhibits the growth of glioblastoma tumor cells and breast cancer cells in vivo [30]. CXCL13 is a chemokine capable of promoting B cell migration [31]. Previous studies have shown that CXCL13 is associated with the prognosis of various cancers including oral squamous cell carcinoma and breast cancer [32, 33]. Over the past few decades, TMEM173 (also known as STING or STING1) was found to play an important role in the production of type I interferons and proinflammatory cytokines. STING1-dependent signaling networks regulate autophagic degradation and different patterns of cell death. Insufficient or overactivation of the STING1 pathway is associated with various pathological conditions, such as tumorigenesis, infection, disseminated intravascular coagulation, autoimmune disease and tissue damage [34]. Recently, TMEM173 was reported to correlate with the clinical status and immune response of HNSCC patients and can be used as a biomarker for improving prognosis [35]. C5orf15 (chromosome 5 open reading frame 15) is predicted to be an integral component of the membrane and haven’t been investigated yet. LIPA (lipase A) functions to catalyze the degradation of low-density lipoproteins to generate free fatty acids and cholesterol. Since hypoxia and hypermetabolism are characteristics of the tumor microenvironment, fatty acid turnover is usually high to meet the requirement of energy and biosynthesis [36]. Lipophagy may play a dual pro- and anti-tumor role. The expression of lysosomal acid lipase (LAL) was suggested to improve lipid metabolism and reduce metastasis in lung and liver cancer [37]. ISG20 is a kind of interferon-induced antiviral exoribonuclease mainly acting on single-strand RNA, and exerts antiviral activity against multiple RNA viruses in an exonuclease-dependent manner [38]. Whereas, high ISG20 expression was found to significantly associated with poor prognosis in liver cancer and clear cell renal cell carcinoma, which was proved to enhance angiogenesis, tumor cell proliferation and metastasis [39, 40].

In our results, the expression of multiple immune checkpoints differed between high-risk and low-risk groups based on our risk model. Blockade of PD1 with nivolumab or pembrolizumab produces durable antitumor efficacy in patients with recurrent or metastatic HNSCC, although only 15–20% of patients respond to treatment [41]. As a PD-1 inhibitor, pembrolizumab can be used in combination with cytotoxic chemotherapy for recurrent or metastatic HNSCC, and a recent clinical trial demonstrated promising clinical activity of pembrolizumab in combination with cetuximab in the treatment of recurrent or metastatic HNSCC [42]. Combined immunotherapy targeting PD-L1 and CTLA-4 has shown enhanced activity in several tumor types. However, further study found no statistically significant difference in OS between durvalumab plus tremelimumab treatment and standard treatment [43]. One study showed that increases in PD-1 and TIM-3 TILs during cetuximab treatment were inversely associated with response in HNSCC patients. Blocking these immune checkpoint receptors may enhance cetuximab-based cancer immunotherapy, potentially improving clinical outcomes in patients with HNSCC [44]. Using the TIDE algorithm, we found that the score of the risk model was significantly positively correlated with the TIDE score. In conclusion, immune checkpoint blockade (ICB) therapy has important value in the treatment of HNSCC, and our risk model has potential value in predicting patient response to it.

In results of immune infiltration analysis, memory B cells, CD8+ T cells, follicular helper T cells, and regulatory T cells were enriched in the low-risk group, while activated dendritic cells and activated mast cells elevated in the high-risk group. CD8+ cytotoxic T cells are capable of releasing granzymes and perforin to directly target tumor cells. Activated CD4+ or CD8+ T cells can also produce anti-tumor cytokines such as IFN-γ to inhibit tumor growth and recruit other immune cells.

It was reported that higher CD8+ tumor infiltrating T-lymphocytes were correlated with improved survival and predicted to be a favorable prognostic factor in HNSCC [45, 46]. Although Tregs are typically immunosuppressive and contribute to the immune escape of tumor, studies found that a high infiltration level of Foxp3+ Tregs was significantly associated with longer survival time of HNSCC patients, which were in accordance with our results [47]. The increased Foxp3+ Tregs in the low-risk group may indicate persistently enhancing immune responses and thereby inhibit tumor progression. The roles of tumor-infiltrating B cells in HNSCC haven’t been clearly elucidated yet since they are so few and excluded in most immune infiltration analysis. A study found that activated, antigen-presenting and memory B cells were enriched in the TME of HNSCC, and further suggested the dual effect of B cells due to their plasticity and heterogeneity [48]. Dendritic cells have been described as a strong antigen-presenting cells (APCs) and to mediate the activation of T cells [49]. However, few studies have explored their roles in HNSCC. The high level of activated dendritic cells in the high-risk group can be related to the attenuated inhibitory effect of Tregs to some extent. Mast cells are widely considered to produce regulatory cytokines targeting various immune cells to participate in anti-infective response, allergy and autoimmunity diseases. Low mast cell density was considered to associated with reduced survival in HNSCC [50].

## Conclusions

We comprehensively analyzed the microenvironment and immune cell infiltration in HNSCC, and further built a nine-gene risk model to explore the prognostic value of immune infiltration-related biomarkers. These findings reveal the pivotal role of tumor microenvironment in HNSCC and can provide new molecular targets for the immunotherapy of HNSCC patients.

## Data Availability

Data analyzed in this study is publicly available from TCGA (expression profiles from the Broad GDAC firehose and phenotypes from the UCSC Xena) and GEO database.

## Abbreviations

APCs: Antigen-presenting cells
ESTIMATE: Malignant Tumors using Expression data
GEPIA: Gene expression profiling interactive analysis
GO: Gene Ontology
GS: Gene significance
GSEA: Gene set enrichment analysis
GSVA: Gene set variation analysis
HNSCC: Head and Neck squamous cell carcinoma
HPA: Human protein atlas
ICB: Immune checkpoint blockade
KEGG: Kyoto Encyclopedia of Genes and Genomes
LASSO: Least absolute shrinkage and selection operator
MM: Module member
SE: Standard error
TIDE: Tumor Immune Dysfunction and Exclusion
TMB: Tumor mutation burden
TME: Tumor microenvironment
TOM: Topological overlap matrix
WGCNA: Weighted gene co-expression network analysis
